# Construction of TiO_2_/WO_3_/TiO_2_ double heterojunction films for excellent electrochromic performance

**DOI:** 10.1038/s41598-024-61911-9

**Published:** 2024-05-20

**Authors:** Zhengqiao Lv, Di Yang, Jianwei Mo, Ziyi Jin, Shuai Chang

**Affiliations:** 1 School of Science, Minzu University of China, Beijing, 100081 China; 2 Department of Materials Science, Shenzhen MSU-BIT University, Shenzhen, China

**Keywords:** Energy science and technology, Materials science, Nanoscience and technology

## Abstract

Electrochromic devices are applied extensively to camouflages, smart windows, heat insulation layers, and automobile rearview mirrors, etc. The amorphous WO_3_ is a very attractive electrochromic material, whereas it suffers from degradation of optical modulation and reversibility on ion exchange owing to those deep trapped ions with irreversible reaction behavior. Herein, we designed and, by using magnetron sputtering, prepared a composite film with TiO_2_/WO_3_/TiO_2_ double heterojunctions, which is capable of eliminating the deep trapped ions by itself under ultraviolet light (UV) assistance. The electrochromic device based on this composite film, after being recovery by short-time UV irradiation, can maintain a high transmission modulation of 94.72% after 7000 cycles of the voltammetry measurement. This feature allows the device to maintain its initial electrochromic performance after prolonged use. Moreover, the double heterojunction structure can reduce colouring time and enormously improve the colouration efficiency (CE) of electrochromic devices. Experimental research shows that when the thickness of the bottom and upper TiO_2_ layer of the WO_3_ film was 145.5 nm and 97.0 nm, respectively, the CE of electrochromic devices reached a perfectly high value (479.3 cm^2^/C), being much higher than that of WO_3_ devices (69.5 cm^2^/C). Functions of the TiO_2_/WO_3_/TiO_2_ double heterojunction in electrochromic device were investigated by combining theoretical analysis and experiment validation, and these results provide a general framework for developing and designing superior electrochromic materials and devices.

## Introduction

The past decades have witnessed rapid development of electrochromic technology^1–5^. Extensive research efforts have been devoted to promoting the application of electrochromism in camouflages, smart windows, heat insulation layers, and automobile rearview mirrors those prevent reflected glare, etc. Particularly, electrochromic devices based on low-cost transition metal oxides (MoO_3_, Nb_2_O_5_, NiO, Cr_2_O_3_, WO_3_) have attracted considerable attention^6–10^. Wherein amorphous WO_3_ is the best known electrochromic material and one of the advance materials entering the smart window market^11,12^. The excellent electrochromic performance and cycle stability of WO_3_ have been demonstrated in early research work. As early as 1975, Giglia reported amorphous WO_3_ films in glycerine-H_2_SO_4_ mixture had a long lifetime of 2 × 10^6^ cycles at 40% transmission modulation^13^. Subsequently, a large amount of study has devoted to basic scientific research and experimental technology research of electrochromic WO_3_. Some representative research results have emerged. Typically, it has been demonstrated that electron injection in the surface states and/or accumulation of electrons in the conduction band can lead to significant color change caused by optical transitions, so increasing the specific surface area of WO_3_ is expected to improve the electrochromic performance. As a typical example, WO_3_ quantum dots in LiClO_4_ electrolyte exhibited a fast switching speed (4.5 s and 4.0 s for colouring and bleaching), a high colouration efficiency (76.8 cm^2^/C) and a long cycle life (10,000 cycles with 10% optical contrast loss)^14^. Again, compared to the individual monometallic systems, the heteronuclear metal can regulate electronic environments and thus could optimize electrochromic activity and stability. TiO_2_ has been proven to be a helpful partner of WO_3_ for improving the electrochromic performance. The reversibility can be improved by adding Ti to WO_3_; the lifetime of TiO_2_-doped WO_3_ thin films can be several times longer than that of pure WO_3_^15,16^. The recently reported WO_3_/TiO_2_ electrochromic devices, being prepared by large-scale manufacture technology, displayed a very high transmission modulation over the wavelength range of 400 nm to 2000 nm under a bias voltage below 0.6 V^17^.

The electrochromic mechanism of transition metal oxides, including WO_3_, is based on the two indispensable activities, i.e. injection/output of electrons and Intercalation/deintercalation of small ions. With regard to electrochromic WO_3_, small ions (such as Li^+^, Na^+^, H^+^, Al^3+^ and so on) insert into WO_3_ matrices during the colouring process^18^. The kinetics of electrochromic colouration has been considered that small ions are localized near W^5+^ color centers, probably by binding to oxygen coordination shells of W^5+^ sites, which is also interpreted as ion trapping. Oppositely, during the bleaching process, small ions are pulled out of the local potential field around W^5+^ sites by applying energy^19^. However, devices based on this electrochromic mechanism have a severe shortcoming, which is that the number of inserted charges is greater than the number of extracted charges during the colouring-bleaching process, and the difference between both usually increases with the number of cycles^17^. The ions that cannot be extracted during bleaching are bound in the electrochromic film. After multiple cycles of color changing, these bound ions seriously deteriorate electrochromic performance. The bound ion is considered as the product of irreversible reactions.

Theories of ion diffusion and relaxation have considered that the host structure contains two types of site: ‘shallow’ sites with low-energy barriers which allows the reversible and fast diffusion of ions throughout the film; ‘deep’ sites surrounded by high-energy barriers where ions become immobile once trapped^19–21^. These bound ions are likely to be the ions trapped in ‘deep’ sites. If these bound ions can escape from ‘deep’ sites, the electrochromic performance will also be restored. This point was supported by of Rui-Tao Wen’s experimental research^19^. Wen et al. applied a higher electric potential on WO_3_ films for 20 h, the electrochromic performance after degradation experiment can be restored to the initial state.

Herein we report a strategy to eliminate bound ions through the device itself in an environment with UV light, thereby disposing of the degradation in electrochromic performance caused by ions accumulation during repeated bleaching-colouring processes. This special function of the device is endowed by the TiO_2_/WO_3_/TiO_2_ double heterojunction. There are literatures report that the complex film of WO_3_/TiO_2_ single heterojunction possesses an excellent electrochromic performance^22,23^. In addition, the use of the WO_3_/TiO_2_ single heterojunction (usually a composite formed by nano-WO_3_ and nano-TiO_2_) as a photocatalytic material can significantly improve photocatalytic efficiency^24,25^. However, the proposed complex film of TiO_2_/WO_3_/TiO_2_ double heterojunction has been rarely reported. The proposed TiO_2_/WO_3_/TiO_2_ film, in which a WO_3_ film was wrapped within TiO_2_ films, reduces the corrosion of electrolyte on WO_3_. Furthermore, the transparent conductive film serving as a charge collecting electrode of the electrochromic device also avoids electrolyte erosion due to its coverage of TiO_2_ film. Importantly, both heterojunctions forming in the TiO_2_/WO_3_/TiO_2_ structure can significantly optimize transmission modulation, colouration rate, and colouration efficiency of electrochromic devices. Moreover, we have demonstrated through theoretical analysis and experimental research that the TiO_2_/WO_3_/TiO_2_ structure can effectively utilize photo-generated electrons of TiO_2_ to reduce the capture of ions at the aforementioned ‘deep’ sites. This feature allows the electrochromic device to maintain its initial performance after prolonged use. To our knowledge, this research work hasn’t been reported yet.

## Theoretical analysis

We conducted a theoretical analysis for the TiO_2_/WO_3_/TiO_2_ double heterojunction based on the energy band of solid-state physics. The Poisson’s equation and the current continuity equations were used for simulating energy band of the TiO_2_/WO_3_/TiO_2_ double heterojunction (details is shown in Supporting Information).

**Figure 1:**
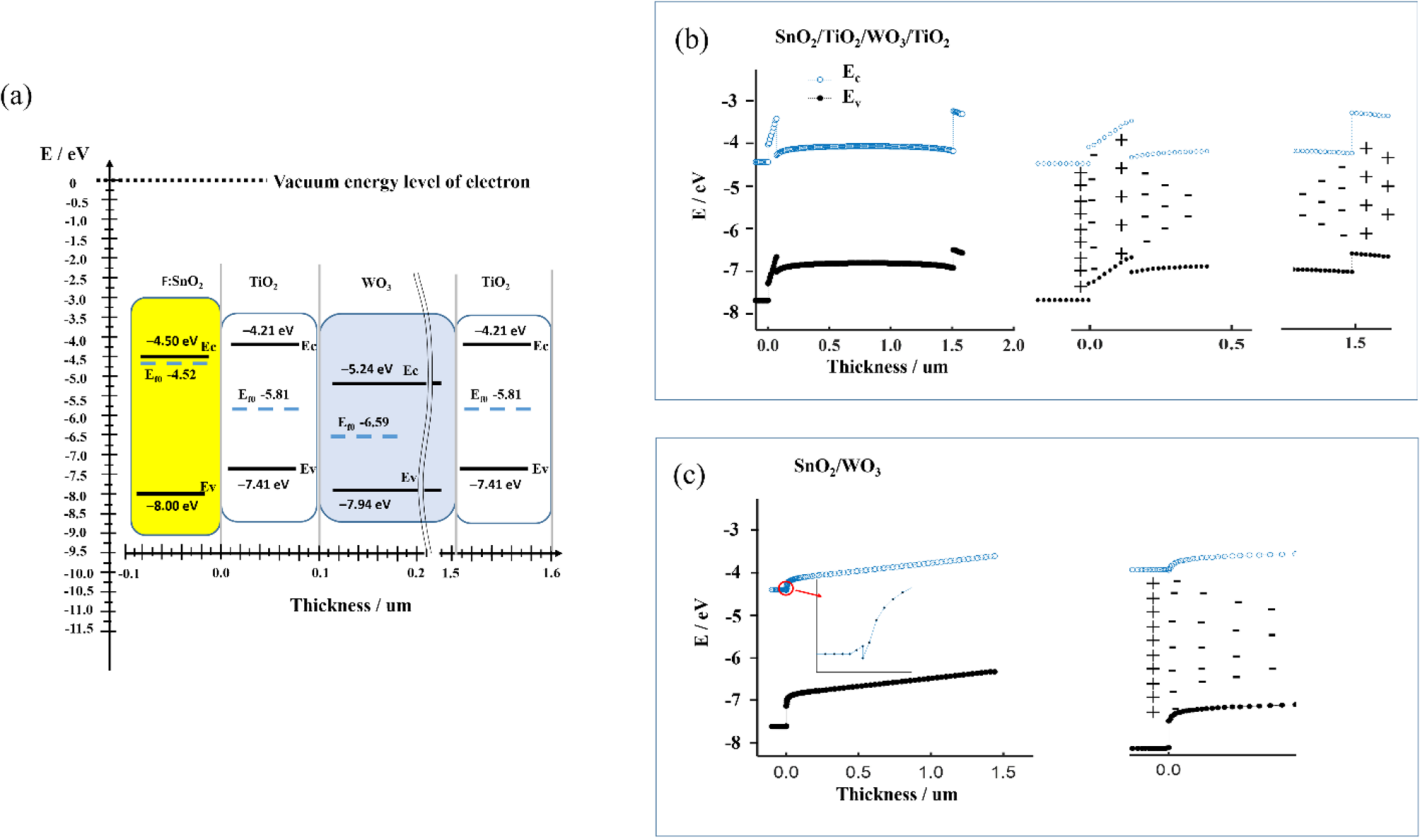
(**a**) A computation model. The x-axis is a gauge of the thickness of each functional layer which was determined by experiments, and the y-axis is energy levels of F:SnO_2_, TiO_2_ and WO_3_ (vs the vacuum energy level of electron) when these materials are separated from each other; (**b**) the energy band as well as the charge distribution of TiO_2_/WO_3_/TiO_2_ on F:SnO_2_ substrate under thermal equilibrium, derived based on the principle of equal Fermi level (E_f_) in a thermal equilibrium system; (**c**) the energy band and the charge distribution of WO_3_ on F:SnO_2_ substrate under thermal equilibrium. The Ec and Ev is bottom of conduction band and top of valence band, respectively.

The computation model was expressed in Fig. 1a, including the important physical parameters of each layer^22^. The difference between TiO_2_ Fermi level (E_f_) and WO_3_ Fermi level is about 0.78 eV^26^ and as well-known, a space charge region (SCR) is formed at the interface between both when they grew together. Based on Poisson’s equation and the current continuity equation, we calculated the energy band diagrams of the thermal equilibrium system of TiO_2_/WO_3_/TiO_2_ on F doped SnO_2_ (F:SnO_2_) substrate by mean of the finite element method, as shown in Fig. 1b. For TiO_2_/WO_3_/TiO_2_ on F:SnO_2_, the bandgap of each layer and the relative position of their Fermi energy levels determine that TiO_2_ layers constitutes potential barriers in the conduction band and potential wells in the valence band. Due to the TiO_2_ work function (~ 5.81 eV) being smaller than the WO_3_ work function (~ 6.59 eV), at the TiO_2_/WO_3_ heterojunction^26^, electrons in TiO_2_ conduction band transfer to WO_3_ conduction band creating a positive charge region on the TiO_2_ side at the interface and meanwhile holes in WO_3_ valence band transfer to TiO_2_ valence band creating a negative charge region on the WO_3_ side. Similarly, at the junction of F:SnO_2_ and TiO_2_, a positive charge region is formed on the F:SnO_2_ side while a negative charge region on the TiO_2_ side. The supposed charge distributions at heterojunctions are also expressed in Fig. 1b.

To compare with TiO_2_/WO_3_/TiO_2_ on F:SnO_2_, we also calculated the energy band diagrams of WO_3_ on F:SnO_2_, which is significantly different from that of TiO_2_/WO_3_/TiO_2_ on F:SnO_2_. In the separated state, the Fermi level of F:SnO_2_ (~ − 4.52 eV) is obviously higher than that of WO_3_ (~ − 6.59 eV) due to that the electron density of F:SnO_2_ is much larger than that of undoped WO_3_. Therefore, when the system of WO_3_ on F:SnO_2_ is in the thermal equilibrium state, the equivalence of Fermi energy levels of various parts results in a higher conduction band bottom of WO_3_ compared to F:SnO_2_. Meanwhile, the as-formed built-in potential is mainly located on the undoped WO_3_ side, but only near the interface on the F:SnO_2_ side, as shown in the insert of Fig. 1c. Figure 1c also exhibits a corresponding charge distribution at the F:SnO_2_/WO_3_ junction.

Comparing TiO_2_/WO_3_/TiO_2_ on F:SnO_2_ and WO_3_ on F:SnO_2_, it is found that TiO_2_/WO_3_/TiO_2_ on F:SnO_2_ has the following advantages in electrochromic performance: (1) when no voltage is applied to the electrochromic electrode, the potential barriers of the conduction band generated by TiO_2_ layers on both sides of WO_3_ not only hinder self-bleaching owing to ion-escape from WO_3_, but also prevent self-colouring caused by fluctuations of environmental potential fields. Therefore the working state of the electrochromic device is more stable; (2) when a negative potential is applied to the electrochromic electrode for colouring, the TiO_2_/WO_3_ junction connected to the F:SnO_2_ substrate is in a positive bias, which reduces the potential barrier and thus is more conducive to injecting electrons into WO_3_. Conversely, the WO_3_/TiO_2_ junction on the other side is in a reverse bias, which is beneficial for positive ion implantation into WO_3_. As a result, the colouration speed is improved; (3) the formation of a SCR can excite WO_3_ colouring at the interface, which reduces the demand of injected charge quantity for the coloured state and thus helps to improve the colouration efficiency of electrochromic devices; (4) more intriguingly, the deeper traps of ions can be eliminated in the TiO_2_/WO_3_/TiO_2_ electrode. In an environment containing ultraviolet light, non-equilibrium carriers are generated in TiO_2_ and WO_3_, and a part of electrons are enriched in the positive potential region of the SCR on the TiO_2_ side, while a part of holes are enriched in the negative potential region of the SCR on the WO_3_ side, causing the system to be non-equilibrium. The local potential field generated by these non-equilibrium carriers enriched in the SCR can promote the transfer of positive charges from WO_3_ to TiO_2_. This means that the local potential field is helpful for those deeper trapped ions to be de-trapped and the good performance of electrochromic film keeps longer. All of these theoretical expected results have been verified by our experiments.

## Experimental method

### Materials

The transparent conducting oxide, fluorine doped tin oxide (F: SnO_2_) in our case, coated glass (FTO) having a sheet resistance of 14 Ω/□ used as substrates for the all experiments. Tungsten targets and titanium targets (99.99% purity, 60 mm diameter and 2 mm thickness) was purchased from Shenyang Baijujie Scientific Instrument Co. Ltd. The anhydrous chloroplatinic acid was from Sigma Aldrich. The electrolyte composed of 0.5 M LiI, 0.5 M 4-tert butylpyridine, and 0.3 M 1,2-dimethyl-3-propylimidazole iodine, as well as a low temperature thermoplastic foil of 60 μm thickness being used to seal the devices were supplied by Wuhan Jingge Solar Energy Technology Co. Ltd.

### Preparation of electrochromic films

Using DC magnetron sputtering technology, the electrochromic films including WO_3_ films or TiO_2_/WO_3_/TiO_2_ composite films were successfully prepared under the optimized preparation condition which was described detailly in our previous article^17^ Herein the important experimental processes are restated.

TiO_2_, WO_3_, and TiO_2_ layers were sequentially deposited on a cleaned FTO glass by DC magnetron sputtering pure metal targets (W and Ti). The effective area of all layers was 2.5 cm^2^. The distance between the target and the substrate was adjusted to be 18 cm. The working chamber was evacuated to 9 × 10^−4^ Pa before sputtering. During the deposition of the films, high purity argon (Ar, 99.99% purity) and high purity oxygen (O_2_, 99.99% purity) were used as the sputtering gas and the reaction gas, respectively. The preparation parameters are summarized in Table 1.

**Table 1:** Summary of DC sputtering parameters.

	Sputtering pressure (Pa)	Sputtering power (W)	O_2_/Ar	Sputtering time
TiO_2_	5.0	100	1:4	1 min to 3 min for TiO_2_ on FTO
				2 min for TiO_2_ on WO_3_
WO_3_	2.0	100	1:4	30 min

The flow rate ratio of O_2_ to Ar gas is a key experimental parameter for preparing electrochromic films. When the oxygen ratio is too low, the resulting films have more oxygen vacancies, which affected the electrochromic performance. According to the evaluation of the electrochromic performance of electrochromic films, the gas flow rate ratio (O_2_/Ar) was selected as 1:4. The optimized sputtering power of WO_3_ films was 100 W. At the same time, in order to tightly integrate TiO_2_ film with the WO_3_ film and reduce the unwanted stress, the sputtering power of TiO_2_ was also set to 100 W. After fixing the parameters of gas flow rate and sputtering power, the thickness of the film depends on the sputtering deposition time. In the light of our experimental research, the WO_3_ film obtained by depositing for 30 min exhibits an excellent electrochromic performance. Similarly, according to our experiment, the deposition time for the TiO_2_ layer on FTO was set to 2 min or 3 min, while the deposition time of the other TiO_2_ layer was set to 2 min.

### Fabrication of electrochromic devices

The electrochromic devices was formed by assembling the as-deposited electrochromic film, a Platinum (Pt) thin film deposited on FTO glass, and the electrolyte into a sandwich type cell, which was sealed with a thermoplastic foil of 60 μm thickness. The structure of the electrochromic device is shown in Fig. S1a. The Pt thin film served as the counter electrode of these devices and was prepared by the spin-coating method which had been described in our previous paper^17^. The Pt-counter electrode and the electrochromic electrode overlapped face-to-face with each other and the areas used for connecting wires were reserved separately. Thermoplastic foils with a width of ~ 1 mm were paved on the edge of devices and heated at 130 °C for 3 min to fully seal electrochromic devices. The thermoplastic foil isolated the upper and lower electrodes as well as formed a cavity for storing electrolytes of the device. A small aperture had been punched by an electric drill at each corner of the counter electrode for injecting electrolyte. A drop of the Li^+^ ion electrolyte was put in the aperture and it was introduced into the cell via vacuum backfilling. The device was placed in a small vacuum chamber to remove air. Exposing it again to ambient pressure causes the electrolyte to be driven into the device. Finally, apertures were sealed by cover glasses and the device assemblage was completed. Moreover, before experimental measurement analysis of electrochromic devices, we conducted several CV tests on the newly packaged devices to ensure stable performance. The names of electrochromic devices based on those different films are shown in Table 2.

**Table 2:** Names of electrochromic devices.

Electrochromic electrode	Names of devices
FTO/WO_3_ (30 min)	ECD1
FTO/TiO_2_ (2 min)/WO_3_ (30 min)/TiO_2_ (2 min)	ECD2
FTO/TiO_2_ (3 min)/WO_3_ (30 min)/TiO_2_ (2 min)	ECD3

The time in parentheses is the depositing time of the layer.

### Characterization

A field emission scanning electron microscope (FE-SEM, Hitachi S-4800, Ltd., Tokyo, Japan) was used for study the surface morphology of the films, whose thickness was measured by using a step profiler (Bruker bektakxt). X-ray diffraction (XRD) measurements were measured on a Bruker/D8 FOCUS X-ray diffractometer (Billerica, MA, USA) with a Cu Kα radiation source (wavelength at 1.5405 Å). The element analysis of electrochromic films was characterized by X-ray photoelectron spectrometer (Thermo Scientific K-Alpha). Optical properties were measured by the Ultraviolet–Visible-near infrared spectrophotometer (UV–Vis-NIR, Lambda950, Perkin). Electrochemical performances were tested by an electrochemical workstation (Zennium, Germany). The recovery test of electrochromic performance was carried out under an ultraviolet (UV) lamp with a wavelength of 365 nm and a power of 200 W. The UV lamp illuminated samples from above, with a distance of 5 mm between both. The light power irradiated on the surface of the tested sample was 20 W.

## Results and discussion

**Figure 2:**
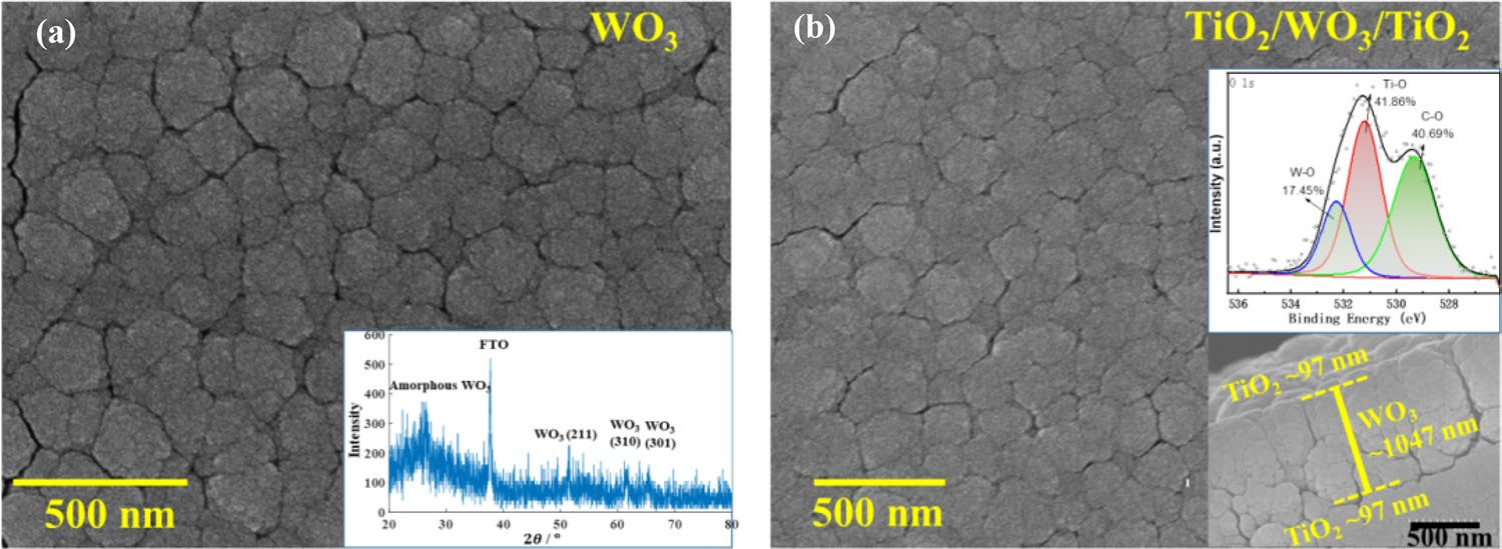
(**a**) SEM micrograph of WO_3_ film and its XRD pattern in the insert map; (**b**) SEM micrograph of TiO_2_/WO_3_/TiO_2_ film attached with insert maps of its cross-sectional view and X-ray photoelectron spectroscopy.

The as-prepared TiO_2_/WO_3_/TiO_2_ composite film was smooth and dense, which can be compared to the WO_3_ film (Fig. 2), implying that the TiO_2_ thin film formed stably and uniformly on the surface of the WO_3_ film. The XRD pattern of WO_3_ complex film revealed the as-deposited WO_3_ film is amorphous (the insert in Fig. 2a). The deposited TiO_2_ film without heat treatment is also amorphous. Due to the TiO_2_ film being too thin to display XRD peak, the X-ray photoelectron spectroscopy (XPS) was used to investigate the chemical composition and existence oxidation state of elements. The insert in Fig. 2b exhibits the peaks of Ti–O (531.2 eV), W–O (532.3 eV) and C–O (529.3 eV) separated from the O 1s spectrum, of which C was an introduced element for calibration. In addition, the cross-sectional view of the composite film is also shown in the insert of Fig. 2b, where the thickness of each layer was demarcated based on measurements using the Step profiler. The thickness of WO_3_ film deposited for 30 min was measured to be 1047.7 nm, and thicknesses of TiO_2_ films obtained by depositing for 2 min and 3 min were measured to be 97.0 nm and 145.5 nm, respectively.

**Figure 3:**
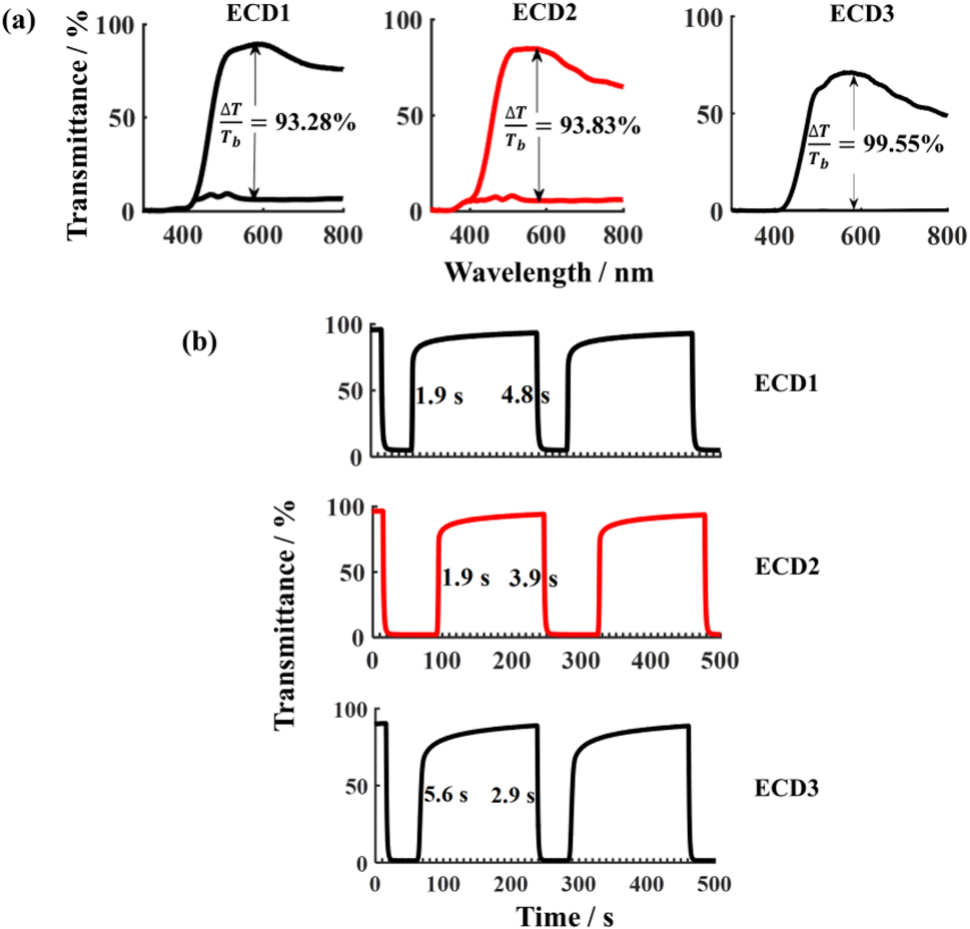
(**a**) Transmittance spectra of ECD1, ECD2 and ECD3, being the bleached state and the coloured state at the bias voltage of 1.0 V and − 1.0 V, respectively. ΔT(T_b_ − T_c_) stands for Transmission modulation, where T_b_ and T_c_ are the transmittance in the bleached and coloured states, respectively; (**b**) bleaching-colouring kinetics measurements of these devices at the wavelength of 580 nm under alternating voltaic cycles from − 1.0 V to 1.0 V.

Figure 3 shows the transmittance spectra and bleaching-colouring kinetics measurements of these devices. After introducing a TiO_2_ coating layer to WO_3_ layer, ECD2 in the bleaching state still has high transmittance, and possesses an ultra-high transmission modulation in the visible light region. For readability, the transmission modulation at 580 nm is marked in Fig. 3a, which is 93.83% for ECD2, slightly higher than that of the WO_3_ device (ECD1). Compared with both ECD1 and ECD2, the transmittance of the bleached ECD3 device clearly decreased. Meanwhile, its transmission modulation reached 99.55%, higher than ECD1 and ECD2, because of its extremely low transmittance in the coloured state.

Bleaching time and coloration time of these devices were revealed by bleaching-colouring kinetics measurements (Fig. 3b). As a reference level to calculate the colouring time or the bleaching time of the device, the highest transmittance in the bleaching state as well as the lowest transmittance in the colouring state of the device were measured. The calculation range of colouring time is the time it takes to decrease from the highest transmittance to 90% of the lowest transmittance. Similarly, the calculation range of bleaching time is the time it takes to increase from the lowest transmittance to 90% of the highest transmittance. Obviously, the colouring time of ECD2 and ECD3 is shorter than that of ECD1, which is consistent with the aforementioned second advantage in the section of Theoretical analysis. However, the bleaching time of ECD3 device increased to 5.6 s, being longer than that of ECD1 as well as ECD2, while the bleaching time of ECD1 and ECD2 is equal. This is also caused by the double heterojunctions. As applying a positive voltage on electrochromic electrode to bleach the device, the TiO_2_/WO_3_ heterojunction connected to the F:SnO_2_ substrate is in reverse bias state, and the electron potential barrier increases, thereby increasing the hindrance force for electron extracting from WO_3_ layer. On the contrary, the WO_3_/TiO_2_ heterojunction on the other side is in a positive bias, and positive charges are easier for extracting from the WO_3_ layer. Therefore, the contribution of both heterojunctions to the bleaching time is opposite. When their contributions cancel out each other, both of heterojunctions have almost no effect on the bleaching time, which is the reason why the colouring time of ECD1 and ECD2 is almost equal. The TiO_2_ layer could not constitute a sufficiently long SCR when its thickness is smaller than the electron diffusion length (such as the 97 nm thickness). In this case, increasing TiO_2_ thickness of the TiO_2_/WO_3_ heterojunction adjacent to the F:SnO_2_ substrate will increase the effectiveness of this heterojunction, leading to an increase in bleaching time. Therefore, ECD3 has a longer bleaching time.

**Figure 4:**
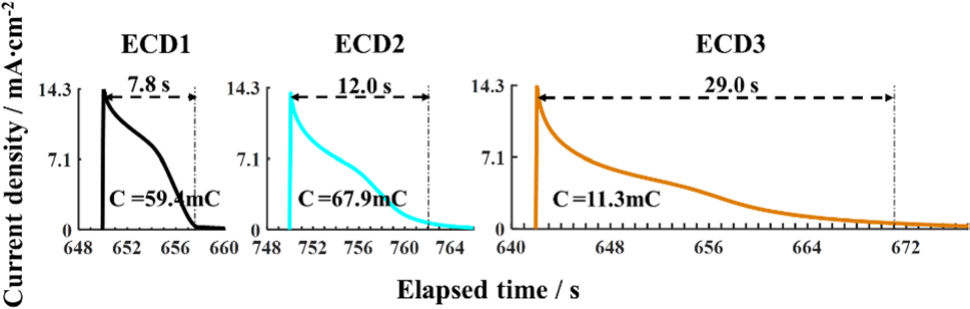
The real-time detecting current curves of these electrochromic devices at an instant of the driving voltage square wave rising from − 1.0 V to 1.0 V.

On the other hand, the dynamic process of the Ionic charge has been regarded to be a main factor affecting the transmission modulation and bleaching-colouring speed of electrochromic devices. The characteristic was investigated by chronopotentiometry module of electrochemical workstation. A voltage square wave from 1 to − 1 V was exerted on the device to make it transform from bleaching to colouring, and both bias voltages held long enough so that Li^+^ ions fully inserted into the electrochromic film and then completely extracted from it. The real-time detecting current of this process expresses quantities of the inserted/extracted Ionic charge as well as ion-transporting time. Figure 4 demonstrates the ion-extracting time and the extracted charge quantity for these different devices. As expected, the thicker the TiO_2_-cladding layer, the longer the Li^+^ transporting time in electrochromic electrode. Interestingly, the Li^+^-ion charge of ECD3 device is much smaller compared to the other devices. In the colouring state, ECD3 device has extremely low transmissivity and fewer inserted Li^+^-ions, demonstrating it possesses an ultra-high colouration efficiency ($CE$), which is usually calculated according to the following formula:

1 $$CE\left(\lambda \right)=\frac{1}{Q}ln\frac{{T}_{b}}{{T}_{c}},$$

where ${T}_{b}$ and ${T}_{c}$ are transmissivities of the electrochromic device in the bleached and coloured states, respectively; the $Q$ is the extracted charge quantity derived from the real-time detecting current curves, as shown in Fig. 4. $CE$s of these devices can be calculated according to Formula (1). Being convenient for analysis, the $CE$s of these devices at the wavelength of 580 nm are listed here, which are 69.5, 41.0, and 479.3 cm^2^/C for ECD1, ECD2 and ECD3, respectively. Obviously, the $CE$ of ECD3 device is much high, almost ten times that of the other devices. This is consistent with the above third advantage obtained from theoretical analysis. During the SCR forming, electrons in TiO_2_ conduction band transfer to WO_3_ conduction band, resulting in a positive charge region on the TiO_2_ side of the interface between both. According to the electrochromic theory of WO_3_, WO_3_ colouring effect occurs while it obtains electrons and the accompanying positive charges. Therefore, the SCR can excite WO_3_ colouring at the interface. It is thus clear that the SCR built by the thickened TiO_2_ layer between F:SnO_2_ and WO_3_ compensates quantity of the inserted Li^+^ ions, and greatly increases the colouration efficiency.

**Figure 5:**
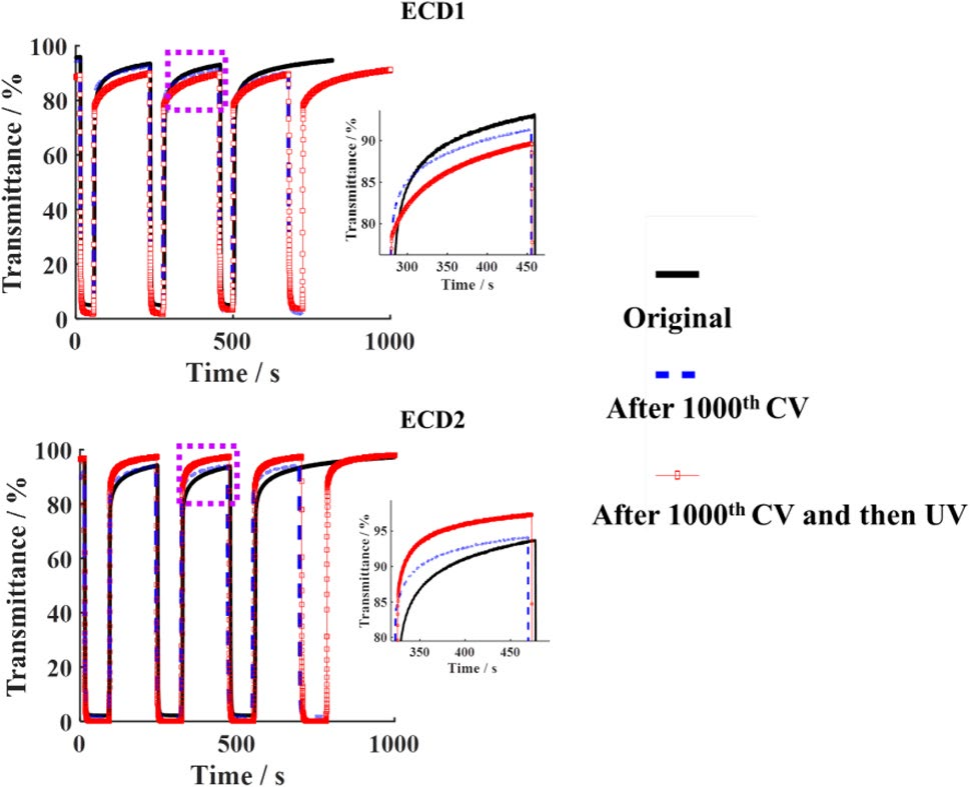
Comparisons of bleaching-colouring kinetics between ECD1 and ECD2. The insert is an enlarged image of the transmittance curve in the bleached state circled by a dashed box. The black solid line, blue dashed line, and dotted red line are the test curves of both devices after 15 CV cycles, after 1000 CV cycles and after 1000 CV cycles, again being irradiated by ultraviolet (UV) light for 15 min, respectively.

More attractively, the electrochromic performance of the device based on the TiO_2_-coated WO_3_ film is restored or even improved after the numerous of cyclic voltammetry (CV) testing and ultraviolet (UV) irradiation. We conducted 1000 cycles of CV testing on ECD2 device and found that its dynamic performance did not decrease but increased. The performance improvement was more significant after 15 min of exposure to the UV lamp. For ECD1 device, however, both 1000 CV tests and ultraviolet light irradiation led to a decrease in the dynamic performance (Fig. 5). The experimental results are consistent with the above theoretical analysis, thereby proving that the TiO_2_/WO_3_/TiO_2_ double heterojunction is helpful for the deeper trapped ions to be de-trapped and improve the device performance.

**Figure 6:**
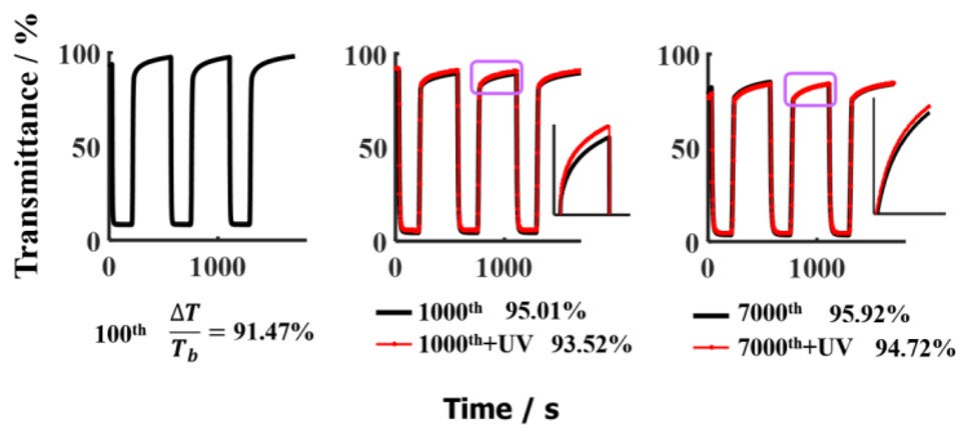
Comparisons of the bleaching-colouring kinetics characteristic of ECD2 after thousands CV measurements and repetitive ultraviolet irradiations. The bias voltage alternated from 1.0 V to − 1.0 V and each UV irradiation lasted for 15 min. The inserts are enlarged images of the transmittance curve in the bleached state circled by boxes.

In replicate experiments, this function of TiO_2_/WO_3_/TiO_2_ double heterojunction is once again demonstrated. Figure 6 records the dynamic characteristics of bleaching and colouring of the electrochromic device at different stages of both CV measurement and UV irradiation. After 7000 CV measurements, the transmission modulation of the device based on the TiO_2_/WO_3_/TiO_2_ still reached 95.92%. Furthermore, the transmittance of the bleached device slightly increases after each exposure to UV light, meaning that UV irradiation is beneficial for the TiO_2_/WO_3_/TiO_2_ device to recover to its initial state.

## Conclusion

In conclusion, the advantages of electrochromic devices based on the TiO_2_/WO_3_/TiO_2_ double heterojunction, including good operational stability, fast colouration rate, high colouration efficiency, and self-elimination of the performance degradation, were derived through theoretical analysis and have been confirmed by our experimental results. The electrochromic device can maintain the transmission modulation of 94.72% after 7000 cycles of the voltammetry measurement and possesses a super high $CE$ of 479.3 cm^2^/C, being much higher than that of WO_3_ devices (69.5 cm^2^/C). Ion trapping is a ubiquitous phenomenon, and therefore the technology that endows the device with ability to eliminate unfavourable trapped charges by itself, thereby maintaining ideal operating state for a long time, is a highly promising key technology. Although the relevance of this technique to practice devices remains to be demonstrated, it may open avenues towards superior smart windows and hence widen the scope for future buildings that are both energy efficient and comfortable for human occupation.

### Supplementary Information

Supplementary Information.

## Data Availability

The original contributions presented in the study are included in the article/Supplementary Material; further inquiries can be directed to the corresponding author.
