# Supplementary material for: Construction of TiO2/WO3/TiO2 double heterojunction films for excellent electrochromic performance
Source: Sci Rep. 2024 May 20;14:11443. doi: 10.1038/s41598-024-61911-9 (PMC11106074; doi:10.1038/s41598-024-61911-9)
Supplement: Supplementary file 1 — Supplementary Information. [file 41598_2024_61911_MOESM1_ESM.docx]

**Construction of TiO_2_/WO_3_/TiO_2_ double heterojunction films for excellent electrochromic performance**

ZhengqiaoLv ‡^a^, Di Yang ‡*^a^, Jianwei Mo ^a^, Ziyi Jin ^a^, Shuai Chang ^b^

a. School of Science, Minzu University of China, Beijing 100081, China. E-mail: diyang@muc.edu.cn， 0000-0001-5292-2021

b. Department of Materials Science, Shenzhen MSU-BIT University, Shenzhen, China. E-mail: schang@smbu.edu.cn, 0000-0001-7120-173X

* Corresponding Author Di Yang

‡Equal contribution

Theoretical method

The energy band diagrams in the thermal equilibrium systems of both TiO_2_/WO_3_/TiO_2_ on F:SnO_2_ and WO_3_ on F:SnO_2_ were calculated by solving basic physical equations in the present model (Figure 1a) through numerical methods. The basic equations are Poisson’s equation and the current continuity equations for electrons and holes. These are expressed in one dimension and in the steady state as^[1]^

$\frac{\mathbf{d}^{\mathbf{2}}\boldsymbol{\phi}}{\mathbf{dx}^{\mathbf{2}}}\mathbf{=}\mathbf{-}\frac{\boldsymbol{q}}{\boldsymbol{\varepsilon}}\mathbf{(}\boldsymbol{p-n+}\boldsymbol{N}_{\boldsymbol{D}}\boldsymbol{-}\boldsymbol{N}_{\boldsymbol{A}}\mathbf{)}$ (S1)

$\frac{\boldsymbol{dJ}_{\boldsymbol{n}}}{\mathbf{dx}}\mathbf{=}\boldsymbol{qR}$ (S2)

$\frac{\boldsymbol{dJ}_{\boldsymbol{n}}}{\mathbf{dx}}\mathbf{=}\mathbf{-}\boldsymbol{qR}$ (S3)

where $\boldsymbol{\phi}$ is the electrostatic potential, $\boldsymbol{q}$ is the electron charge,$\boldsymbol{\varepsilon}$ is the dielectric permittivity, $\boldsymbol{p}$ and$\mathbf{n}$ are hole and electron densities, respectively, $\boldsymbol{N}_{\boldsymbol{D}}$ and $\boldsymbol{N}_{\boldsymbol{A}}$ are the ionized donor and acceptor densities, respectively,and $\boldsymbol{J}_{\boldsymbol{n}}$ and $\boldsymbol{J}_{\boldsymbol{p}}$ are the electron and hole current densities, respectively. According to Shockley-Read-Hall statistics,$\mathbf{R}$ represents the recombination rate and is expressed as

$\boldsymbol{R}\boldsymbol{=}\frac{\boldsymbol{pn}\boldsymbol{-}\boldsymbol{n}_{\boldsymbol{i}}^{\boldsymbol{2}}}{\boldsymbol{\tau}_{\boldsymbol{n}\boldsymbol{0}}\left( \boldsymbol{p+}\boldsymbol{n}_{\boldsymbol{i}} \right)\boldsymbol{+}\boldsymbol{\tau}_{\boldsymbol{p}\boldsymbol{0}}\left( \boldsymbol{n+}\boldsymbol{n}_{\boldsymbol{i}} \right)}$ (S4)

where $\boldsymbol{n}_{\boldsymbol{i}}$ is the intrinsic carrier density and $\boldsymbol{\tau}_{\boldsymbol{n}\boldsymbol{0}}$ and $\boldsymbol{\tau}_{\boldsymbol{p}\boldsymbol{0}}$ are the electron and hole lifetimes, respectively. In the bulk region, excluding the heterojunction interface, the current equations are expressed as

$\boldsymbol{J}_{\boldsymbol{n}}\boldsymbol{=}\boldsymbol{\mu}_{\boldsymbol{n}}\boldsymbol{n}\frac{\boldsymbol{d}\boldsymbol{E}_{\boldsymbol{Fn}}}{\boldsymbol{d}\mathbf{x}}$ (S5)

$\boldsymbol{J}_{\boldsymbol{p}}\boldsymbol{=}\boldsymbol{\mu}_{\boldsymbol{p}}\boldsymbol{p}\frac{\boldsymbol{d}\boldsymbol{E}_{\boldsymbol{Fp}}}{\boldsymbol{d}\mathbf{x}}$ (S6)

where $\boldsymbol{\mu}_{\boldsymbol{n}}$ and $\boldsymbol{\mu}_{\boldsymbol{p}}$ are the electron and hole mobilities, respectively, and $\boldsymbol{E}_{\boldsymbol{Fn}}$ and $\boldsymbol{E}_{\boldsymbol{Fp}}$ are the electron the hole quasi- Femi levels, respectively.

The material parameters used in this calculation are presented in Table S1. Band parameters and optical parameters such as energy bandgaps, electron affinities, relative permittivities, etc. are taken from the reported references.^[2-5]^

Table S1 Material parameters used in this calculation

| Property | F:SnO_2_ | TiO_2_ | WO_3_ |
| --- | --- | --- | --- |
| Electron mobility (cm^2^/(Vs)) | 37.8 | 0.1 | 83.3 |
| Relative permittivity | 3.61 | 4.84 | 5.29 |
| Band gap (eV) | 3.50 | 3.20 | 2.70 |
| Electron affinity (eV) | 4.50 | 4.21 | 5.14 |


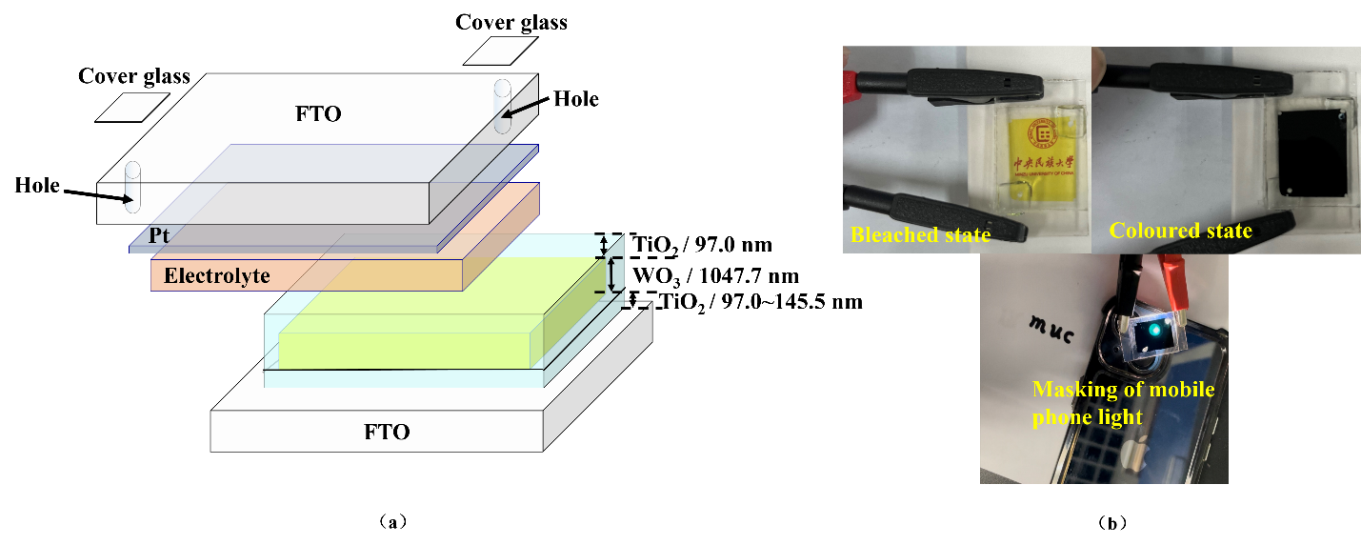


Figure S1.(a) Configuration scheme of the electrochromic device; (b) photographs of the TiO_2_(146nm)/WO_3_/TiO_2_ device in the bleached and coloured states.

Reference

1. *Horio, K.; Yanai, H., Numerical modeling of heterojunctions including the heterojunction interface. IEEE Transactions on Electron Devices* **1990***, 37 (4), 1093-1098.*
2. *Akagawa, M.; Fujiwara, H., Optical characterization of textured SnO2:F layers using spectroscopic ellipsometry. Journal of Applied Physics* **2012***, 112 (8), 457.*
3. *Banyamin, Z.; Kelly, P.; West, G.; Boardman, J., Electrical and Optical Properties of Fluorine Doped Tin Oxide Thin Films Prepared by Magnetron Sputtering. Coatings* **2014***, 4 (4), 732-746.*
4. *Yamada, Y.; Kanemitsu, Y., Determination of electron and hole lifetimes of rutile and anataseTiO_2_ single crystals. Applied Physics Letters* **2012***, 101 (13), 37.*
5. *Lee, Y. A.; Han, S. I.; Rhee, H.; Seo, H., Correlation between excited d-orbital electron lifetime in polaron dynamics and coloration of WO_3_ upon ultraviolet exposure. Applied Surface Science* **2018***, 440 (MAY15), 1244-1251.*
